# 

**Published:** 1895-04

**Authors:** 


### CONTENTS.

ORIGINAL COMMUNICATIONS—
The History of Medicine and Surgery in Georgia. By Luther B. Grandy, M.D., Atlanta, Ga. .......... 65
A Case of Onychogryphosis with Cornu Cutaneum. By W. H. Inghram, M.D., Atlanta, Ga. .......... 75
Contusions and Sprains of the Back, with Special Reference to the Early Treatment of these Injuries. By Henry R. Wharton, M.D., Philadelphia .......... 79
The Administration of Anesthetics. By Jas. B. Morgan, M.D., Augusta, Ga. .......... 85
Some Remarks on Gunshot Wounds. By H. McHatton, M.D., Macon, Ga. .......... 89
SOCIETY REPORTS—
Richmond Academy of Medicine and Surgery .......... 93
CORRESPONDENCE—
Sage Tea for Hiccough .......... 100
EDITORIAL—
The Medical Association .......... 102
Commencements .......... 104
SELECTIONS AND ABSTRACTS—
Skin Diseases and Syphilis .......... 106
Eye, Ear, Nose, and Throat .......... 108
Surgery .......... 113
Obstetrics and Gynecology .......... 117
BOOK REVIEWS .......... 122
MEDICAL ITEMS .......... 127
ADVERTISERS' INDEX TO ADVERTISEMENTS .......... xvi
CLASSIFIED ADVERTISING INDEX .......... xiv
MEDICAL ITEMS—Continued .......... ix, x, xi, xii, xiii, xv

